# Outcome of Selective Laser Trabeculoplasty in Young Patients with Primary Open-Angle Glaucoma and Ocular Hypertension

**DOI:** 10.1155/2020/5742832

**Published:** 2020-06-09

**Authors:** Dan Liu, Di Chen, Qian Tan, Xiaobo Xia, Haibo Jiang, Jian Jiang

**Affiliations:** ^1^Eye Center of Xiangya Hospital, Central South University, 87 Xiangya Road, Changsha, China; ^2^Hunan Key Laboratory of Ophthalmology, 87 Xiangya Road, Changsha, China; ^3^Ophthalmology of the Second People's Hospital of Foshan, 78 Weiguo Road, Foshan, China

## Abstract

**Purpose:**

To determine the effectiveness and safety of selective laser trabeculoplasty (SLT) in young patients with primary open-angle glaucoma (POAG) and ocular hypertension (OHT).

**Methods:**

This was a retrospective clinical study. Fifty-six eyes from 56 young (age ≤ 40 y) patients with POAG or OHT treated with SLT were included. According to age, patients were divided into group 1 and group 2. Patients in group 1 were younger than 18 years old, and patients in group 2 were between 18 and 40 years old. Patients were evaluated before treatment and at 1 hour, 1 day, 1 week, 1 month, 3 months, 6 months, and 1 year after treatment. We also collected older patients (age ≥ 60 y) who received SLT during the same period for comparison at 1-year follow-up. Possible factors affecting the success of SLT, including baseline IOP, age, sex, diagnosis (POAG or OHT), and whether or not use of antiglaucoma medication before treatment, were analyzed.

**Results:**

SLT treatment produced significant reduction in IOP in the young patients with POAG or OHT during the 1-year follow-up period（*P* < 0.05）. Mean IOP at 1 hour after SLT was lower in group 1 than in group 2 (*P* < 0.01), but at other follow-up time points, IOP values were not different (*P* > 0.05). IOP reduction and success rate were not significantly different between young and old patients at 1 year after treatment. IOP measurements over a 24-hour period were recorded before and after the SLT in 20 young adult patients. IOP values were significantly lower in the treated patients at all time points than at pretreatment (*P* < 0.05), and 24-hour mean IOP, peak IOP, valley IOP, and fluctuation in IOP were also lower in SLT-treated patients (*P* < 0.05). Baseline IOP was found as a predictor of SLT success in young patients (OR = 1.895, *P*=0.003), whereas age, gender, diagnosis, and whether or not use of antiglaucoma medication were not correlated with SLT success (*P*=0.725, *P*=0.750, *P*=0.061, and *P*=0.201, respectively).

**Conclusion:**

In this study, SLT was found as an effective and safe treatment for young patients with POAG and OHT. High baseline IOP predicted high SLT success.

## 1. Introduction

Selective laser trabeculoplasty (SLT) was introduced in 1995 [1] and received United States Food and Drug Administration approval in 2001. The procedure has become an established method for lowering intraocular pressure (IOP) in open-angle glaucoma (OAG) and ocular hypertension (OHT) [2, 3]. SLT uses a Q-switched, frequency-doubled, neodymium:yttrium-aluminum-garnet (Nd:YAG) laser, which emits at 532 nm. The laser selectively targets pigmented trabecular cells, thereby increasing aqueous outflow through the trabecular meshwork without causing thermal damage to adjacent nonpigmented meshwork structures. Clinical trials for SLT have been encouraging, with reasonable response rates, moderate reduction in IOP, and minimal side effects [2–9]. Consequently, SLT was recently designated as first-line treatment for OAG and OHT by the Laser in Glaucoma and Ocular Hypertension Trial [4, 5].

However, all studies of SLT have included older patients. In previous studies, the mean age was more than 60 years old (Table 1). To our knowledge, only one investigation focused on the efficacy of SLT in younger patients; however, the patients in their study were individuals under the age of 60 years. Thus, few studies have evaluated the efficacy of SLT in young OHT and juvenile glaucoma patients, and little is known about the efficiency and safety of SLT for these patients. Since some studies have suggested that ALT is statistically less effective in younger patients than in older patients [17, 18], it is not known if the effect of SLT in young patients is different from that in older patients. In our study, we retrospectively collected data on 56 eyes from 56 young patients with POAG or OHT who underwent SLT in our hospital. The main aim was evaluation of the ocular hypotensive effect of SLT. The second goal was assessment of the predictive factors of success after SLT treatment in these young patients.

## 2. Materials and Methods

### 2.1. Patients and Study Population

This was a retrospective chart review. We defined patients younger than 40 years of age as young. Young POAG and OHT patients treated with SLT from January 2016 to December 2017 in the ophthalmology clinic of Xiangya Hospital, Central South University, China, were included. The study was conducted in accordance with the ethical principles specified in the Declaration of Helsinki and approved by the Xiangya Ethics Committee. All patients or guardians signed consent for SLT treatment, and data were collected anonymously. IOP was measured before treatment and at 1 hour, 1 day, 7 days, 1 month, 3 months, 6 months, and 12 months after treatment.

Patients were consecutively introduced into the study. Inclusion criteria were POAG or OHT patients that were younger than 40 years and had received SLT treatment. Exclusion criteria were as follows: (1) previous SLT or incisional ocular surgery; (2) eye disease other than POAG or OHT; (3) follow-up for less than 1 year; and (4) two or more missed IOP measurements. If both eyes were eligible for study, both were treated with SLT, but data from only one eye were chosen randomly for analysis.

Young patients included in our study were divided into group 1 and group 2 according to age. Patients in group 1 were younger than 18 years old, and patients in group 2 were between 18 and 40 years old. For comparison, we also evaluated elderly patients (age ≥ 60 y) who received SLT during the same period.

Pretreatment examinations consisted of determination of the best corrected visual acuity, slit lamp examination, gonioscopy, Goldmann applanation tonometry with three measurements taken in the afternoon (3 : 00pm), fundus examination, optical coherence tomography (OCT) of the optic nerve head, white-on-white standard automated perimetry performed using a Humphrey Visual Field Analyzer (Zeiss-Humphrey Systems, Dublin, CA) with the C-24-2 SITA standard strategy, and 24-hour IOP measured at 10am, 2pm, 6pm, 10pm, 5am (the next day), and 7am (the next day). Data recorded from each patient included age, sex, type of glaucoma, antiglaucoma medications, baseline IOP, 24-hour IOP, and SLT protocol (number of spots and laser power settings). IOP was reexamined at 1-hour, 1-day, 7-day, 1-month, 3-month, 6-month, and 12-month follow-ups. 24-hour IOP was monitored again at 6-month follow-up, and gonioscopy, fundus examination, visual field, and OCT were reexamined at 12-month follow-up.

All SLT procedures were performed by the author Dan Liu, using a Q-switched Nd:YAG laser (Ellex Solo Ellex Medical Pty. Ltd., Adelaide, Australia) with topical anesthesia and a Latina Lens (Ocular Instruments Inc., WA, USA). Treatments were performed on 180° of the trabecular meshwork, either inferior or superior. The laser was used at a standardized setting of spot size 400 *μ*m, power range of 0.8 to 1.2 mJ, and pulse duration of 3 ns. Initial power setting was 0.8 mJ. Fine bubbles were sought for each laser application, and the power of the laser was increased to achieve bubbles at each application. Fifty-five spots were applied to the trabecular meshwork side by side. No hormone or nonsteroidal anti-inflammatory eye drops were used after SLT treatment. Patients who had used antiglaucoma drugs before SLT treatment used the same drugs after treatment. A second SLT was performed when the patient's IOP was not decreased by at least 2 mm Hg at 1-month follow-up.

Complications, such as conjunctival congestion, flashing of the anterior chamber, transient elevation of IOP, and corneal epithelial injury, were evaluated at 1 hour, 1 day, and 7 days after treatment.

Data were extracted from patient records at the baseline visit (pretreatment) and at follow-up visits (posttreatment). Successful treatment was defined as a reduction of more than 20% in IOP with no change in pharmaceutical treatment or additional surgery needed at 1-year follow-up. Glaucoma staging was performed according to the modified Hodapp–Anderson–Parrish glaucoma classification based on visual field status [19]. Mean deviation (MD) values ＞−6 dB were classified as early glaucoma, values between −6 dB and −12 dB were classified as moderate glaucoma, and values ＞−12 dB were classified as advanced glaucoma. IOP values at each follow-up visit were compared with baseline IOP, and values for the two patient groups were compared. At 1-year follow-up, the success rate and reduction in IOP in young patients were compared with the elderly patient group. Possible factors affecting the success of SLT, including baseline IOP, age, sex, whether or not use of antiglaucoma medication, and diagnosis (OHT or POAG), were assessed.

### 2.2. Statistical Analysis

Student's *t*-test for paired data was used to assess the changes in IOP from baseline values and differences in IOP between the age groups. Success rate between young and elderly patients at 1 year after treatment was assessed with Fisher's exact test. Repeated measures ANOVA analysis was used to compare the mean IOP between different time points in the young patients. To determine the possible factors affecting the success of SLT, univariate and multivariate logistic regression analysis was used. Multivariate logistic regression analysis was performed for variables with *P* < 0.10 on univariate analysis. The results were considered statistically significant with values of *P* < 0.05.

## 3. Results

Fifty-six eyes from 56 young patients (18 patients were younger than 18 years old and 38 patients were at age between 18 and 40) were included in this study. Twenty-three eyes from 23 elderly patients (≥60 years) were collected for comparison with eyes from younger patients at 1-year follow-up. Baseline characteristics of the three groups are listed in Table 2.

### 3.1. IOP-Lowering Effect

In young patients, one patient received surgery and one patient increased the use of antiglaucoma drugs at 6 month follow-up because of the increase of IOP. These two patients were classified as “failure,” but their original data were used. Four patients received a second SLT treatment 1 month after the first SLT treatment.

**Figure 1:**
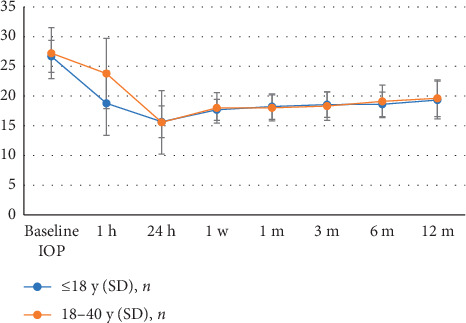
Mean IOP at various time points up to 1 year.

SLT treatment resulted in significant IOP reductions in all young patients during 1-year follow-up (*P* < 0.05).  Figure 1 displays the mean IOP values of the patients in the two groups during the entire follow-up period. The mean IOP in both groups started to decrease 1 hour after treatment, decreased to the lowest value at day 1, and stabilized after day 7. After SLT, IOP at day 1 was lower than at any other follow-up points (*P* < 0.05), and there was no significant difference among IOP recordings at 7 d, 1 m, 3 m, 6 m, and 12 m follow-up (*P* > 0.05). Mean IOP at 1 hour after SLT was significantly lower in group 1 than in group 2 (*P*=0.003), but IOP in the two groups was not significantly different at later points (*P* > 0.05).

### 3.2. IOP in Young Patients versus Old Patients

IOP values at baseline and 1 year after SLT, as well as the reduction of IOP in the two age groups, are illustrated in Table 3. Baseline values were significantly higher in young patients (*P*=0.02), but values at 1 year were not significantly different (*P*=0.59). The reduction in IOP values for the two groups after 1 year was also not significantly different (*P*=0.13). The success rate of 71.4% (40/56) in young patients vs 56.5% (13/23) in old patients was not significantly different (*P*=0.20).

### 3.3. 24-Hour IOP

**Figure 2:**
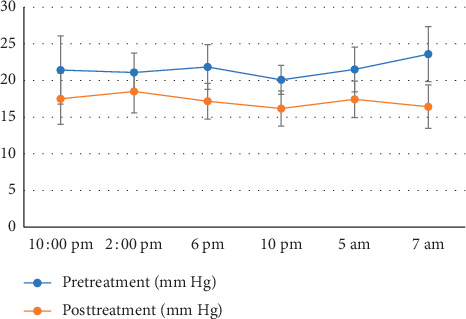
Twenty-four-hour IOP before and 6 months after SLT treatment in 20 young patients.

Twenty-four-hour IOP was measured at pretreatment and at 6 months after treatment in 20 young patients (Figure 2). The IOP at 10am, 2pm, 6pm, 10pm, 5am, and 7am was significantly lower than at the pretreatment time point (*P* < 0.05). The 24-hour mean IOP value, peak value (maximum value of 6 time points), valley value (minimum value of 6 time points), and fluctuation value also were significantly lower at 6 months than before SLT treatment (*P* < 0.05) (Table 4).

### 3.4. Visual Fields

Visual field was examined in 56 young patients and 18 old patients before treatment and reexamined in 50 young patients and 15 old patients at 1-year follow-up time. The visual field were all still normal in all OHT patients at 1 year after surgery. Mean MD 1 year after surgery in POAG patients did not show a statistical difference compared to before surgery with 6.4 ± 4.8 dB and 6.8 ± 3.0 dB, respectively (*P*=0.10). However, 2 of 35 POAG patients show obvious visual field progression, which showed that the ranges of scotomas were enlarged.

### 3.5. Possible Factors Affecting the Success of SLT

The results of univariate and multivariate logistic regression analysis of possible factors affecting success rate of SLT in young patients with POAG or OHT are illustrated in Table 5. On univariate logistic analysis, patients' diagnosis (POAG or OHT) and baseline IOP were significantly correlated with success of SLT (*P*=0.04, *P* < 0.001), while multivariate logistic analysis showed that only baseline IOP was significantly associated with the success of SLT (*P*=0.001).

### 3.6. Complications

Transient IOP elevation occurred in 14 eyes (26.4%) 1 h after SLT, of which 5 eyes (9.4%) had IOP elevation >5 mm Hg and 9 eyes (17.0%) had elevation < 5 mm Hg. No transient IOP elevation >8 mm Hg or IOP ≥40 mm Hg occurred. Thirteen eyes (24.5%) had conjunctival hyperemia, 25 eyes (47.2%) had mild anterior-chamber flashing, and 6 eyes (11.3%) had epithelial punctate keratitis that disappeared at 24 h after treatment. One year after operation, gonioscopy found no scar formation or anterior synechia in the iridocorneal angle.

## 4. Discussion

To our knowledge, this is the first study to focus on the outcome of SLT treatment for young patients who have POAG or OHT. We retrospectively collected 56 eyes from 56 young POAG or OHT patients undergoing SLT and found that SLT effectively lowered IOP in this population. At 1-year follow-up, treatment resulted in 8.22 mm Hg (30.4%) IOP reduction; the success rate was 71.4%. We also compared IOP reduction and success rate between young patients and old patients at 1 year after treatment and found no difference between the two groups, suggesting that SLT can reduce IOP in young patients as well as in older patients. In previous studies, mean IOP reduction ranged from 16.9% to 31.9% with a success rate of 58% to 94% at 1-year follow-up [2, 9, 20–23]. Our results are consistent with these studies, in which the average patient age was >60 years old.

The mean IOP in young and old patients started to decrease at 1 h after SLT treatment; the lowest value occurred at day 1 and then stabilized after day 7. This result showed that IOP values at 7 days after treatment are likely predictive of future IOP control; this is consistent with that of other reports [24, 25].

Since patients younger than 18 years old were excluded in other reports on SLT, we deliberately included this population (18 patients) in this study. IOP was lower in these patients than in patients with age 18–40 at 1 h after SLT, but values of the two groups were not significantly different at other time points. Thus, SLT was as effective in lowering IOP in patients younger than 18 years old.

Lowering IOP can prevent the progression of glaucoma, but fluctuations of IOP are also a risk factor for progression. The Advanced Glaucoma Intervention Study reported that long-term fluctuations of IOP are associated with progressive loss of the visual field [26, 27]. Thus, in-office recordings of IOP should not be the sole measure of treatment effectiveness of SLT; reduction of IOP fluctuation is also an objective. In previous studies [28–30], SLT decreased the amplitude of diurnal IOP fluctuation significantly in older patients. We compared IOP over a 24-h period in 20 young patients before SLT and 6 months after SLT; mean, peak, trough, and fluctuation of IOP values were consistently and significantly lower after operation than before operation. Our results indicate that SLT is also effective in controlling IOP fluctuation in young patients.

Reported complications associated with SLT are usually mild, transient, and self-limited [31]. In this study, adverse effects, such as redness, anterior-chamber inflammation, and transient increase in IOP, occurred within 1 h after SLT, but these phenomena disappeared without treatment within 24 h. One year after operation, gonioscopy found no scar formation or anterior synechia in the iridocorneal angle, indicating that SLT caused no obvious damage to the trabecular meshwork. Thus, SLT appears to be safe in young patients.

It is difficult to establish definite, robust predictors of SLT success, and multiple studies have had various results. However, the most consistently reported patient factor predictive of success is elevated baseline IOP [2, 3, 11]; other patient factors, such as sex, race, age, glaucoma type, trabecular meshwork pigmentation, lens status, and central corneal thickness, have not been found to be predictive of success. Similarly, we found that higher pretreatment baseline IOP predicted higher 1-year success rates with SLT. By contrast, age, type of glaucoma, and use of pretreatment antiglaucoma medication did not.

Our study was limited by its retrospective design, which may have affected the selection of patients. For example, in our hospital, most patients with advanced glaucoma receive antiglaucoma surgery instead of SLT, so few advanced glaucoma patients were included in our study, although the severity of glaucoma may be a predictor factor. Another limitation in our study was that the follow-up data up to 6 months after treatment in elderly patients were incomplete; thus, we could not compare the IOP at all follow-up times between young and old patients. As a result, we evaluated the short-term results (i.e., 1 year), but results in the long term may differ from those in the short term.

## 5. Conclusions

Our study identified selective laser trabeculoplasty as an effective and safe treatment for young patients with primary open-angle glaucoma and ocular hypertension. The treatment can reduce intraocular pressure and pressure fluctuations for these patients and can be used as primary or adjunct therapy. High pretreatment intraocular pressure is a predictor of success with selective laser trabeculoplasty.

## Figures and Tables

**Table 1:** Mean patient age in prior SLT studies.

Principal author		Mean patient age (year)
Damji et al. [10]		69.7 ± 10.52
Prasad et al. [11]		67 ± 10
Gazzard et al. [5]		63.4 ± 12.0
Hutnik et al. [12]		64.95 ± 10.60
Garg et al. [13]		63.4 ± 12.1
Lee et al. [14]		60.30 ± 16.20
Kurysheva et al. [15]		70 ± 7.39
Gazzard et al. [4]		64 ± 12
Liu et al. [16]		48.7 ± 9.4

**Table 2:** Baseline characteristics of patients.

	≤18 years	18–40 years	≥60 years
No.	18	38	23
Age (year)	12.3 ± 2.3	32.3 ± 2.6	32.3 ± 2.6
	(11–17 y)	(11–17 y)	(11–17 y)
Female/male	8/10	12/26	10/13
No. of POAG	6	20	12
No. of OHT	12	18	11
No. of antiglaucoma medication			
0	18	16	12
1	0	18	8
2	0	4	3
Type of antiglaucoma medication			
Timolol	0	10	5
Prostaglandin	0	16	7
Brimonidine	0	0	2
Stage of glaucoma			
Early	3	8	3
Moderate	3	10	7
Advanced	0	2	2

POAG, primary open-angle glaucoma; OHT, ocular hypertension.

**Table 3:** IOP and success rate at 1-year follow-up in young and elderly patients.

	Young patients	Elderly patients	*P*
No	56	23	
Baseline IOP (mm hg)	27.05 ± 3.57	24.63 ± 4.35	0.02^*∗*^
IOP at 1 year (mm hg)	18.83 ± 3.12	18.42 ± 2.09	0.59
IOP reduction (mm hg)	8.22 ± 3.11 (30.4%)	6.21 ± 2.09 (25.2%)	0.13
Success rate	71.4% (40/56)	56.5% (13/23)	0.20

**Table 4:** The 24-hour mean IOP value, peak value, valley value, and fluctuation value before and after SLT treatment.

	Mean	Peak	Valley	Fluctuation
Pretreatment (mm hg)	21.58 ± 2.00	25.33 ± 3.09	18.17 ± 1.70	7.17 ± 2.79
Posttreatment (mm hg)	17.19 ± 2.54	19.08 ± 3.00	15.33 ± 2.57	3.75 ± 1.29
*P*	＜0.001^*∗*^	＜0.001^*∗*^	0.003^*∗*^	＜0.001^*∗*^

^*∗*^Statistically significant.

**Table 5:** Univariate and multivariate logistic regression analysis of possible factors affecting SLT success in young POAG or OHT patients.

Covariates	Univariate logistic analysis			Multivariate logistic analysis		
	OR	*P*	95% confidence interval	OR	*P*	95% confidence interval
Diagnosis (OHT)	3.02	0.040^*∗*^	0.948–8.021	0.975	0.061	0.702–24.455
Age (y)	0.974	0.105	0.944–1.006	1.015	0.570	0.946–1.070
Gender (male)	0.333	0.106	0.079–1.150	0.598	0.589	0.092–3.799
Baseline IOP (mm Hg)	1.844	＜0.001^*∗*^	1.319–2.591	2.236	0.001^*∗*^	1.409–3.761
Anti-glaucoma medication (yes)	1.030	0.956	0.335–2.702	—	—	—

IOP, intraocular pressure; POAG, primary open-angle glaucoma; OHT, ocular hypertension. ^*∗*^Statistically significant.

## Data Availability

The data used to support the findings of this study are included within the article.
